# Pain Beliefs in the Military: A Qualitative Study Exploring the Perspectives of Instructors Within the Canadian Armed Forces on How Pain Is Addressed in Basic Training and the Broader Military Culture

**DOI:** 10.1080/24740527.2026.2682925

**Published:** 2026-07-28

**Authors:** Mael Gagnon-Mailhot, Peter Stilwell, M. Gabrielle Pagé, Derek Speirs, Hélène Le Scelleur, Timothy H. Wideman

**Affiliations:** a Department of Psychology, Université de Montréal, Montreal, Quebec, Canada; b Centre de recherche du Centre hospitalier de l’Université de Montréal (CRCHUM), Montreal, Quebec, Canada; c School of Physical and Occupational Therapy, McGill University, Montreal, Quebec, Canada; d Department of Psychology, University of Southern Denmark, Odense, Denmark; e Department of Anesthesiology and Pain Medicine, Université de Montréal, Montreal, Quebec, Canada; f Patient partner, Sherbrooke, Canada; g School of Social Services, University of Ottawa, Ottawa, Ontario, Canada; h Chronic Pain Centre of Excellence for Canadian Veterans, Hamilton, Ontario, Canada; i Centre for Interdisciplinary Research in Rehabilitation of Greater Montreal (CRIR), IURDPM, CIUSSS-Centre-Sud-de-l’Ile-de-Montréal, Montreal, Quebec, Canada

**Keywords:** Veterans, pain beliefs, chronic pain, military basic training, injury stigma, military culture

## Abstract

**Background:**

Pain beliefs play an important role in how people understand and cope with pain, and they are especially relevant for military personnel and Veterans who bear a disproportionately high burden of chronic pain. However, limited research has examined which pain beliefs are central to military training and the broader military culture from the perspective of active basic training instructors.

**Aim:**

The current study aims to explore what beliefs about pain are emphasized in basic military training and the broader military culture from the perspective of active basic training instructors within the Canadian Armed Forces.

**Methods:**

We used a qualitative descriptive study design involving two semi-structured focus groups with instructors. The transcripts were analyzed using inductive content analysis.

**Results:**

Three themes were generated. Theme 1 describes how instructors aim to teach recruits to prioritize mission and team success by pushing through pain and distinguishing it from injury. Theme 2 outlines the culture around pain in training and the broader military. Injuries are often experienced as personal failures and threaten one’s identity. Thus, there is pressure to endure painful injuries and stigma for seeking care. Theme 3 describes instructors’ critical reflections on how pain is addressed within the military, emphasizing that the military’s relationship with pain is a double-edged sword with both helpful and harmful aspects.

**Conclusions:**

There is a tension between the military’s operational goals and the effective management of pain and injuries. Implications for military training and future research are discussed.

## RÉSUMÉ

**Contexte :** Les croyances relatives à la douleur jouent un rôle important dans la façon dont les personnes comprennent la douleur et y font face. Elles sont particulièrement pertinentes pour le personnel militaire et les anciens combattants, chez qui le fardeau associé à la douleur chronique est disproportionnellement élevé. Toutefois, peu d’études ont examiné les croyances relatives à la douleur qui occupent une place centrale dans la formation militaire et, plus largement, dans la culture militaire, du point de vue des instructeurs en service actif responsables de la formation de base.

**Objectif :** La présente étude vise à explorer les croyances relatives à la douleur mises de l’avant dans la formation militaire de base et, plus largement, dans la culture militaire, du point de vue d’instructeurs en service actif responsables de cette formation au sein des Forces armées canadiennes.

**Méthodes :** Nous avons utilisé un devis descriptif qualitatif comprenant deux groupes de discussion semi-structurés avec des instructeurs. Les transcriptions ont été analysées au moyen d’une analyse de contenu inductive.

**Résultats :** Trois thèmes ont été dégagés. Le thème 1 décrit comment les instructeurs cherchent à enseigner aux recrues à donner la priorité à la réussite de la mission et de l’équipe en surmontant la douleur et en la distinguant d’une blessure. Le thème 2 présente la culture entourant la douleur dans le cadre de la formation et, plus largement, au sein de l’armée. Les blessures sont souvent vécues comme des échecs personnels et menacent l’identité de l’individu. Il en résulte une pression qui pousse à endurer des blessures douloureuses ainsi qu’une stigmatisation associée au fait de demander des soins. Le thème 3 décrit les réflexions critiques des instructeurs sur la façon dont la douleur est abordée au sein de l’armée, en soulignant que le rapport de l’armée à la douleur constitue une arme à double tranchant, comportant à la fois des aspects bénéfiques et néfastes.

**Conclusions :** Il existe une tension entre les objectifs opérationnels de l’armée et la prise en charge efficace de la douleur et des blessures. Les implications pour la formation militaire et les recherches futures sont abordées.

## Introduction

In Canada, one in five people lives with chronic pain, a condition that costs the nation an estimated $40 billion per year.^1^ Canadian Veterans experience nearly twice the prevalence of chronic pain compared to the general population.^2^ Military personnel with combat injuries experience the highest rates of chronic pain.^3^ A range of biopsychosocial factors influence chronic pain among Veterans, such as demographics (age, sex, education, relationship status), service-related characteristics (duties, rank, deployment), and mental health disorders (major depressive episode, generalized anxiety disorder, panic disorder, and posttraumatic stress disorder (PTSD)).^4^ However, little is known about the potential origins of how Veterans experience and manage persistent pain during and after service. As new recruits first enter the Canadian Armed Forces (CAF), their basic training (Basic Military Qualification (BMQ)) aims to help them form a military state of mind,^5^ also referred to as the military identity.^6–10^ The military identity encompasses prominent service-related beliefs, attitudes and values that form a sense of community and shape an individual’s self-identity.^6,9^ It is possible that beliefs about pain, first emphasized by basic training instructors during BMQ, are influential in shaping the military identity and culture around pain.

Pain beliefs are an important part of self-identity and can be defined as how an individual understands their own pain, as well as the pain of others, and what they should do when they are in pain (i.e., how they should cope).^11^ These beliefs are shaped by biological, psychological, and social factors, such as gender, socioeconomic status, learning experiences, spirituality, intergenerational trauma, family history in the military, family history of pain and pain beliefs, as well as genetic and epigenetic influences.^12–16^ For instance, those who believe that their pain is an indicator of damage to their body are more likely to disengage from valued activities and life roles.^17,18^ In other cases, those whose self-identity is anchored to productivity, resilience, and perfectionism may strive to ignore their pain and try to relentlessly push through it.^19^ Thus, pain beliefs can impact coping, including engagement with self-management and activity-based interventions.^18,20^ The military identity likely encompasses specific pain beliefs that help service members prioritize the successful completion of missions regardless of personal discomfort, which could also exacerbate the tendency to push beyond limits.^21^ For example, the motto “pain is weakness leaving the body”^22^ and similar variations are commonly used in the military to help members form the belief that they should push through pain and “suck it up”^23(p299)^. However, this same belief and associated behavior may be problematic,^21^ especially during transition to civilian life.^23^ For Veterans living with chronic pain, pain beliefs associated with a strong military identity may undermine uptake of important evidence-based pain-management strategies, such as pacing engagement in pain-related physical activity to prevent disabling flare-ups.^23^ Common messages in civilian healthcare settings (e.g., activities should be paced out) may not be in line with the pain beliefs that were developed by Veterans over the course of their military career.

A recent study with military personnel living with chronic pain suggests that pain beliefs considerably influence psychological well-being, physical health, and quality of life.^24^ Another study highlighted the importance of taking into account the pain beliefs and cultural background of military personnel to effectively treat Veterans living with chronic pain.^25^ Despite pain beliefs often being alluded to or addressed through standard questionnaires in the scientific literature,^11,24,26–35^ there is currently very limited research that directly explores which pain beliefs basic training instructors emphasize during the BMQ course. Recently, a meta-synthesis on the impact of the military lifestyle on the health of current and former service members with chronic pain called for qualitative research to explore the complex interactions between identity, culture, gender, mind-set, purpose, and pain in the military.^36^ As a starting point to help fill these gaps, the current study was designed to explore what beliefs about pain are emphasized by instructors in the initial formation of the military identity and how these relate to the broader culture of the CAF.

## Methods

### Study design

We used a qualitative descriptive study design involving semi-structured focus groups.^37,38^ This approach is appropriate for obtaining a straightforward description of phenomena known to a group; in our case, beliefs about pain that are emphasized in the initial formation of the military identity. Our guiding paradigm was naturalism with aspects of interpretivism and constructivism, as described by Sandelowski.^39,40^ Sandelowski^39,40^ argues that naturalism is the typical foundation for qualitative descriptive studies. She operationalizes naturalism as a commitment to trying to understand a phenomenon in its natural state. With respect to focus groups, this commitment means striving to achieve descriptions that reflect participants’ actual practices. Sandelowski^39,40^ outlines ways to obtain such descriptions, including refraining from using a specific a priori theory or manipulating variables that might distort participants’ descriptions. Because of this naturalistic positioning, she argues that qualitative descriptive studies are the least theoretical of the available qualitative approaches. However, she emphasizes that this does not render qualitative descriptive studies atheoretical, nor does it imply an absence of researcher influence. Elements of interpretivism and constructivism remain at play, as researchers inevitably impart some degree of interpretation and influence throughout the research process. She states: “Although no description is free of interpretation, basic or fundamental qualitative description, as opposed to, for example, phenomenological or grounded theory description, entails a kind of interpretation that is low-inference, or likely to result in easier consensus among researchers”^39(p335)^.

Throughout the research process, we utilized a participatory approach engaging Veterans living with chronic pain (D.S., H.L.S. and two others).^41^ These Veterans living with chronic pain were recruited through the Quebec Pain Research Network (QPRN).^42^ For more details on the involvement of the Veterans living with chronic pain, see the GRIPP2 Short Form in the supplemental digital content section of this article.^43^ Briefly, they were involved in the conception of the research project and participated in every stage of the research, primarily through their engagement in team meetings where they discussed and gave feedback on elements, such as recruitment procedures, focus group guide, and interpretation of the findings. The involvement of individuals with lived experiences is in accordance with established pain research recommendations.^1,44,45^ To support rigorous reporting, we followed the Consolidated Criteria for Reporting Qualitative Research (COREQ) and the Guidance for Reporting Involvement of Patients and the Public (GRIPP2 Short Form).^43,46^ Ethics approval was granted by the Research Ethics Office at McGill University’s Faculty of Medicine and Health Sciences (A03B3121B) and informed consent was obtained from all participants. For further details, including reporting checklists, please refer to the supplemental digital content.

### Study setting and participants

Active basic training instructors leading the nine-week BMQ course (which aims to integrate civilians into the military and teach them the basic skills and mind-set required to become members of the CAF)^47^ were recruited from the Canadian Forces Leadership and Recruit School (Quebec). The focus groups were conducted in a private room at the Canadian Forces Leadership and Recruit School, with all participants seated around a table.

### Recruitment

We used a convenience sampling method. A staff member at the school shared a study recruitment document and connected the research team with interested instructors. This document framed the study as an inquiry into the pain beliefs that are central to military identity and how recruits are taught to think about, understand, and manage their pain. Basic training instructors who expressed interest in participating were provided with a link to an online information letter/consent form (created and administered via LimeSurvey-McGill University).

### Sample size and justification

Based on anticipated *information power* and guidance on focus group sampling,^37,48,49^ we aimed to recruit two groups with four to eight instructors per focus group (one Anglophone and one Francophone group). Smaller focus groups can be preferable because they allow participants to feel more comfortable sharing their thoughts on complex topics, such as the one presented in our study.^50^ The literature suggests that two or three focus groups are sufficient to generate a satisfactory number of themes in qualitative research.^49^ These criteria were used alongside information power to inform our sample size a priori.^48^ The concept of information power suggests that when a sample provides rich and relevant data for the study, fewer participants are required. This was assessed using the grid presented in the supplemental digital content.^48^ Following Malterud and collaborators’ information power criteria,^48^ we judged a relatively small sample sufficient: the aim was narrowly defined, the sample was specific (basic training instructors), participant–researcher dialogue was strong, and the analysis targeted one defined group rather than between group comparisons. Information power also guided our assessment of the sample size during data collection, indicating that we had gathered enough data, as participants were open and shared rich, detailed, and deeply personal experiences, which further increased the information power of the sample.

### Data collection

Instructors completed the consent form prior to the focus group. Those consenting to participate were presented with a set of demographic questions. Some of this information is kept confidential to protect the participants’ identities. We included two language groups, as participants did not share a common language and data collection had to be conducted separately in each language. The focus groups were conducted in-person at the Canadian Forces Leadership and Recruit School (Quebec) during the winter of 2024. The focus groups were led by T.W. and G.P., who followed a semi-structured focus group guide. This guide was developed in collaboration with Veterans (D.S. and two others) and shaped by their experiences with basic training and military culture. Questions focused on identifying messages that instructors aim to instill in recruits regarding how they should understand their pain and what they should do when they are in pain. The focus group guide included questions such as “Can you please tell us more about the military state of mind and behaviour that you are trying to cultivate among new recruits?”, “How do you or your colleagues shape new recruits’ understandings of pain?” and “What happens when someone has a painful injury, and it is not safe to continue to serve?” (for the complete focus group guide, please refer to the supplemental digital content). The focus groups were audio-recorded and M.G.M. took field notes during the focus groups. Focus groups were well-suited for our purposes as they enable group dynamics that can help uncover shared cultural practices and norms within a group.^37,38^

### Data analysis

The recordings were transcribed and translated (French to English) by a hired professional and then reviewed by M.G.M., T.W. and P.S. independently. M.G.M. conducted an inductive content analysis of the transcripts.^51,52^ Content analysis is often chosen in qualitative descriptive studies because it provides a low-inference approach to data analysis and allows codes to be generated directly from the data.^39^ Coding was conducted in MAXQDA^53^ to generate preliminary themes that summarized the data. M.G.M. presented the coded data and these early themes to T.W. and P.S. during weekly meetings held throughout the analysis process. To support rigor, we used investigator triangulation, with T.W. and P.S. regularly reviewing the developing codebook and thematic structure and providing feedback that was incorporated.^54^ Themes and supporting quotations were presented to D.S. and H.L.S. for feedback and to discuss alignment with their lived experience in basic training and service in the CAF. M.G.M. reviewed the field notes taken during the focus groups to ensure that any important insights and reflections were incorporated into the coding scheme and the themes that were generated.

## Results

We recruited a total of eight participants who were male and identified as men, with an average age of 37.4 years. Participants had an average of 15.8 years of service. Five identified as White and three identified as belonging to other ethnic groups (this is reported as “others” to protect the anonymity of the participants). Nationalities included five Canadians and three participants from other nationalities (this is reported as “others” to protect the anonymity of the participants). Each focus group, lasting 2 hours each, had four participants. Information power criteria^48^ were used to assess the sample size and to confirm that we had gathered sufficient information to achieve the objective of our study. No participants withdrew from the study, and no data were excluded.

We identified three core themes, each with subthemes, which cover various aspects of pain beliefs within the context of military training and culture. The first theme focuses on the core objectives for what new recruits need to learn about pain. The second theme examines the culture surrounding pain in basic training and within the broader military environment. The third theme involves critical reflections on how pain is addressed within training and the military as a whole. Co-authors who are Veterans living with pain (D.S. and H.L.S.) indicated that the themes, analysis and conclusions closely aligned with their experiences in the Canadian military.

Below, we discuss these themes and their associated subthemes in greater detail, with supporting quotations from the two focus groups. Note that when quotes are reported, the participant number and the group to which they belong are listed. When no focus group number is listed, this indicates a direct reply to the quoted participant above.

### Theme 1: core objectives for what new recruits need to learn about pain

This theme focuses on what instructors identified as the explicit goals of their teaching regarding pain beliefs during basic training. According to the instructors, mission success is of paramount importance in the military. Thus, it is important for recruits to learn to distinguish between pain and injuries. This learning enables military personnel to push through pain when necessary to complete missions and to seek medical treatment when an injury occurs.

These findings from Theme 1 are explored in greater depth in the three subthemes: (1.1) recruits must learn to prioritize mission and team success; (1.2) recruits must learn to push through pain; and (1.3) recruits must learn to distinguish between pain and injuries.

#### Recruits must learn to prioritize mission and team success

As context for the reader, CAF military personnel operate under the principle of unlimited liability. According to the Department of National Defence, this principle means that “in the process of their duties, they may have to risk their lives or the lives of those they lead to achieve success in the military missions assigned by the Government of Canada.”^55^ Accordingly, instructors highlight the fact that personal well-being is seen as secondary during missions.

Participant 3 (Group 1): *There’s no other option when it comes to the mission right? … if someone got hurt? Like – well injured and really injured we still have to do our job. Right? So, if you’re just hurt? We don’t necessarily need to hear about it. Just keep going. Let’s get the mission done.*

Instructors mention that during missions, individual discomfort must be set aside in favor of the team’s collective needs to ensure mission success. They emphasize that enduring physical challenges together fosters a sense of unity and strengthens team cohesion.

Participant 4 (Group 1): *… the mission aspect is instilled in your brain. You have to get the job done regardless. If you’re hurt, everyone’s hurt. And that’s the thing, if everyone is in the hurt, and everyone is enduring the suck together? That cohesion is automatically instilled. Like OK, yeah I’m hurting. Everyone is, top to bottom, let’s go.*

#### Recruits must learn to push through pain

As seen in the previous subtheme, ensuring mission success requires military personnel to remain operational, even when facing severe difficulties. One of the key ways military personnel can achieve this is by pushing through pain and discomfort, which is why instructors aim to teach recruits this skill and reward this behavior.

Participant 1 (Group 2): *… it’s really about rewarding the effort. When you see that it’s been tough, when doing a good job hasn’t been easy, then it’s about recognizing it. What you went through wasn’t easy, but you succeeded. That gives them the confidence to say, “I succeed in pain.” Create patterns like that.*

Ensuring mission success is achieved by getting recruits out of their comfort zones and exposing them to physical and mental stressors in an effort to build resilience.

Participant 3 (Group 2): *Physical training is how you develop resilience. We also develop resilience when we’re outdoors. When the weather’s really bad, we let the elements take over. … pouring rain for days or you’re cold, well … Cold, freezing, you know. Hot, freezing, hot, freezing, that’s the worst.*

The instructors also aim to lead by example and demonstrate their ability to push their own limits by enduring pain during grueling physical workouts.

Participant 3 (Group 1): *I have fitness staff that will push them through their workouts. I try to go and show an example of what it should look like. So that if I look in that much pain, then you should probably look like this at the end of a workout. Right? But it’s just exposure really in the grand scheme of things, and getting these people to do it. Provide some motivation, push limits.*

Instructors report that teaching by example not only sets a standard but also inspires recruits to persevere and to be proud of pushing themselves, which further supports recruits in pushing through their limits.

Participant 2 (Group 2): *I built them up from the bottom slowly … I knew I wasn’t going to hurt anyone because we’d been training from the start. By the end, everyone was proud of themselves … Resiliency comes with the territory. And so does shape. I tried to give them the pleasure of training.*

Instructors note that this desired behavior of pushing beyond limits is reflected in pain-related slogans yelled by the recruits when going through difficult times. Instructors sometimes promote those slogans themselves and feel that they help build team cohesion and reinforce beliefs about pain that they want to instill. These slogans are centered around the idea that pain is something that should be pushed through.

Participant 1 (Group 1): *Oh yeah, “embrace the suck” here is pretty common. I’ve seen that one around here quite a bit.*

Participant 4 (Group 1): *Well obviously in the field. It’s raining, everyone’s on the verge of hypothermia. Just embrace the suck is what I say. Suck it up. Suck it up, Buttercup.*

Participant 3 (Group 2): *No pain, no gain.*

#### Recruits must learn to distinguish between pain and injuries

Pain is considered a desirable aspect of training, but instructors emphasize that injuries are not. Hence, it is important for the instructors to teach recruits to make that distinction.

Participant 1 (Group 2): *We want pain, but we don’t want injury.*

Participant 2 (Group 1): *… (we are) trying to explain the difference between being hurt (in pain) and being injured.*

Participant 4 (Group 2): *… (recruits) don’t understand the difference between pain related to an injury or just pain because you’ve been training, or good pain.*

Instructors note that recruits’ lack of experience with exercise and recovery is a challenge that must be addressed. Instructors aim to teach that exercise-related pain signifies recovery and muscle growth, rather than an injury requiring medical attention.

Participant 4 (Group 2): *That’s what they have the hardest time with. Accepting pain. Often, it’s not even, as you say, an injury. It’s just discomfort. Or I often get some that are just sore. You’ve trained, then you’re out the next day. Hello, goodbye. They want to go to the hospital because they’re aching all over. Because you don’t understand, you’ve never trained, it’s your body that’s getting stronger. You’ve got to keep going, it’s going to hurt, start your day. Then, after an hour, or an hour and a half of your day, you will be moving better.*

### Theme 2: culture around pain in basic training and the broader military

This theme highlights aspects of the broader military culture related to beliefs about pain that influence basic training but are not part of the core objectives outlined in the previous theme. Instructors discussed how military culture shapes one’s personal identity, beliefs and behaviors in relation to pain and injury. Further, they discussed how military culture permeated basic training and their own service experiences.

The following two subthemes unpack the influence of military culture on pain-related beliefs: (2.1) injuries are experienced as personal failures that threaten one’s identity and (2.2) military personnel often feel pressured to endure painful injuries, as seeking medical care is stigmatized.

#### Injuries are experienced as personal failures that threaten one’s identity

Instructors reported that during basic training and active military service, there is a strong connection between failure and injury, as being injured often means having to retake a course. Thus, military personnel greatly fear injuries as they often mean failing courses.

Participant 4 (Group 1): *I would say 90% of the failures or re-coursed (being removed from the current course and placed onto another course at a later point) is because of injury. … Because they don’t want to fail. They don’t want to get re-coursed. They want to pass and move on. That’s their biggest fear. Which is pretty much everyone, every military personnel that’s being assessed. That’s our biggest fear. Don’t get injured.*

This fear of injury is rooted in the demoralizing prospect of having to restart training and being left behind by the team, something that is often perceived as having failed.

Participant 1 (Group 1): *But one thing that works against the whole teamwork atmosphere that we emphasize on the first couple weeks. Oh, you guys are a team, you’re a team. Now you’re not part of the team. You’re going to watch your peers graduate. Because you’re not going to graduate on that time. And you’re going to watch them, and you might come back in two, three, four, six months and join a whole new group. And you’re now going to have to start all over.*

Instructors suggest that being reliable is often deeply ingrained in one’s sense of identity and pride. This aspect of the military identity can lead individuals to act as if they were on missions during training and to push through injuries to avoid letting the team down or being perceived as weak.

Participant 2 (Group 1): … *I knew I was severely hurt … and then I was like well it’s fine, we’re on a “mission.” And I just fell back into that mind-set, right? I was like I’m on a “mission.” I’m not going down, I’ll just finish it (the training session). And then I’ll go to medical once everything’s done … All the instructors knew I was hurting … And it’s like I’m not getting in the safety vehicle, no. The candidates are here, I’m doing the “mission” with the candidates. I’m just hurt, it’s OK.*

Participant 4: *That’s the pride thing. We’re all, like proud.*

Instructors observe that military personnel often push through both pain and injuries because mission success is so central to their identity. In the previous theme, we saw that instructors view pushing through pain as essential for mission success. However, this mind-set can lead to unintended consequences, as military personnel may also push through injuries.

Participant 3 (Group 1): *A lot of the people that would be struggling with these things (injuries) would be the people that kept going. Kept fighting through pain. Identity, like their entire identity is about carrying out the mission. Keep going … Do it no matter what.*

As mentioned in the previous theme regarding instructors teaching by example when pushing through pain, instructors also unintentionally convey to recruits that working through injuries and remaining operational are inherent parts of military life, which contributes to disabling injuries being perceived as a form of failure.

Participant 1 (Group 2): *It’s about setting an example, showing them that we’re capable of being in pain, we have aches and pains, things happen. In the field, they see me limping, it’s normal, I have injuries that will follow me all my life. So, they see me, but then they see me running. Then I limp a little. They see it. They see it, then they look. I explain to them that such and such a thing happened, but I’m able to get through it, because I need to.*

Participant 2 (Group 1): *I’ll do all of their fitness classes with them. So that I’m there showing them, like you said. If I can do it, you can do it. And just to joke around with them, I always tell them if I’m able to do this work out. And I’ve been in the infantry … years with shitty knees and all broken. Like you’re just joining at 18? You should be able to crush them right? So, I try to motivate them through example.*

#### Military personnel often feel pressured to endure painful injuries, as seeking medical care is stigmatized

To build on the last subtheme, not only are injuries seen as a source of failure and a threat to identity, but military personnel are often pressured to endure injuries and avoid the medical system. As discussed in subtheme 1.3 “recruits must learn to distinguish between pain and injuries,” training strongly emphasizes teaching the difference between pain and injuries. This distinction between pain and injuries aims to help military personnel push through pain while avoiding the risks associated with pushing through injuries. However, instructors revealed that there is often pressure within the military culture to push through injuries as well. According to the instructors, this pressure to endure injuries stems from the importance of the mission and the feeling of guilt when letting their peers or unit down, which can take priority over themselves and their health.

Participant 2 (Group 1): *… understanding that if you are injured yes, that’s something that we will deal with when we have the chance to deal with it. Because the mission comes first.*

Instructors note that this pressure to endure injuries also comes from peers, as injuries that limit military personnel’s ability to work are often perceived negatively due to the additional workload they create. This pressure fosters a general atmosphere where military personnel feel compelled to perform despite their injuries to avoid feelings of guilt associated with letting their peers or unit down.

Participant 4 (Group 2): *When you go to the hospital and come back … let’s say, five days you can’t work. Well, you go to your boss’s office … he’ll look at you. “Are you serious? We need you this week, and now you’re telling me it’s five days.” He turns to someone else. That someone else is your buddy next door. “Well, you’re going to work Monday-Tuesday, you’re going to work Wednesday-Thursday-Friday” because the other buddy here is sick he can’t do it. “OK, you’re hurt, well, look. It’s someone else who’s going to be forced to cover for you.”*

Participant 1 (Group 2): *You’re frowned upon. And then, well, don’t take the time off, it’s not good for your career. Hey, don’t do that, think of others. You’re there and then you say yeah, yeah, yeah. I’ve got to think of others again. The others all the time.*

This pressure becomes internalized and experienced as guilt, as military personnel often push themselves through injuries to avoid increasing the workload on others.

Participant 2 (Group 2): *… My back hurts. At one point, I was doing my duty, and was unable to get up, sleeping on the bed here or in the ditch. But I didn’t want to give up, because if I did, someone would have to take my shift.*

The instructors describe a mentality where admitting to injuries or seeking medical assistance is viewed as a sign of weakness and can be judged negatively by others, particularly when injuries are not apparent.

Participant 4 (Group 2): *That’s it. The bone has to come out or it bleeds. Otherwise you’re getting judged.*

Participant 3: *Yes, that’s it. You just don’t want to get judged … Even in the recruit course we didn’t want to (get judged).*

Instructors also end up contributing to the pressure to perform despite injuries when emphasizing to recruits that they should avoid getting medically checked when in pain and that they should push as long as possible before getting checked for potential injuries.

Participant 4 (Group 2): *During the day, let’s say, at noon, if you hurt yourself somewhere, unless it’s really an emergency, it’ll be postponed to the next day. Some people are able to drag their injury into the next day. At that point, if you can get through the day, why do you have to go to hospital the next morning? As far as I’m concerned, you can do another one. Then, once you’ve done the other, you’ll be able to do another.*

As discussed in the previous subthemes, the threat of missing training periods that could lead to being re-coursed is always looming. This threat can be exacerbated when recruits consider seeking medical help for potential injuries, as instructors often mention that taking medical leave could cause recruits to fail their course, which adds further pressure to avoid seeking care when in pain.

Participant 4 (Group 2): *I often look at the schedule with them. The schedule, let’s say, for the week or the schedule for the day, and then the appointment. If you go there (seeking assessment/care), you’ll spend at least three hours, you’ll miss all these periods. And those periods can’t be rescheduled. You have to understand that if you miss those periods, you risk dropping out of your course. (They are like) “Oh yeah, well no, it’s fine, it’s okay, I won’t go …*”

To further illustrate that recruits avoid disclosing injuries to avoid being re-coursed, instructors mention that most recruits tend to wait until their course is completed before seeking medical treatment. This delay in injury disclosure results in recruits seeking much needed care only when it is least disruptive to their progression through the course and at times when they won’t feel pressure from the group to be operational.

Participant 4 (Group 1): *But the injuries really start to pop up during the field training, field portion of the course. Or the final exercise. Like the ones who really want to stay here and have that mental resilience? They’ll push, push, push until the X is finished. And then you’ll see “OK, we need to go to MIR (medical inspection room).” Or we tell them “Hey, go to the MIR.”*

### Theme 3: critical reflections on how pain is addressed within training and the broader military

Pain beliefs in the military are shaped by a culture of resilience, which has traditionally discouraged seeking medical care. However, instructors have noted a shift toward greater consideration of personal experiences and greater encouragement of the use of medical services. While this shift reflects progress, the military’s relationship with pain remains complex, as it strives to find a balance between building the resilience needed for mission success and the potential long-term harm of untreated injuries. Instructors also highlighted that the separation between the instructors and the medical system within the military creates additional barriers to achieving this balance, as the two systems may have conflicting goals and communicate poorly.

We explore the above in greater depth in three subthemes: (3.1) the military has grown more accommodating of personal experiences and pain, (3.2) the military’s relationship with pain is a double-edged sword and (3.3) the organization of medical care within the military is a barrier to finding solutions.

#### The military has grown more accommodating of personal experiences and pain

Despite certain aspects of the culture favoring military personnel pushing through injuries, as discussed in the previous theme, instructors also note that, over time, there has been a shift toward open discussions in military culture about how injuries and pain are managed. In the past, recruits were expected to endure pain silently and only seek medical help when absolutely necessary.

Participant 3 (Group 2): *In my day, first of all, you didn’t walk (unassisted) to the MIR. It’s another matter of resiliency, because on days like today (during a snow storm), you had crutches, a wheelchair. You’d walk (with crutches) or be wheeled by someone to the MIR. Storm or no storm, that was it.*

Instructors report that discussing pain has become more accepted, and space is made to listen to and reassure recruits when they express discomfort.

Participant 1 (Group 2): *Yes. It’s also that they’re often worried. It’s new for them, they’re afraid. It’s scaring them. They just need to be listened to and reassured, sometimes. … “OK, the sergeant understands what I’m going through, OK.” That’s when they feel heard. Normally, that calms them down.*

According to the instructors, in previous generations, engaging with the medical system was once almost universally perceived as a sign of weakness. Nowadays, the instructors are more accepting of recruits using the medical system and even encourage its use.

Participant 2 (Group 1): *… we encourage candidates to go to the medical system, right? If they’re hurt? Or sorry if they’re injured. And I think that that was something that wasn’t a thing when I went through basic (training). It was very much, especially a combat arms mind-set of like, if you’re going to the medical system it’s because you’re weak. Where I think that mind-set’s like, at least at the instructor level here at the school has changed. Where OK, these are the services provided to you. You are entitled to use them. You just have to ask us. If you don’t ask us, we can’t help you.*

Military personnel now have access to services that promote recovery which are integrated into the training courses.

Participant 2 (Group 1): *The school as a whole is doing a lot more to support … classes have mobility and recovery (training practices focused on improving joint flexibility, range of motion, and helping the body recover between workouts (e.g., stretching, foam rolling, rest, or active recovery)). Which is fantastic, and it’s something that is super important in all aspects of training. Whether it’s civilian side if you’re doing CrossFit, or whatever. Mobility and recovery is a critical part to your plan.*

Instructors report that an important part of changing the mind-set is to make recruits aware that taking care of an injury will make them more reliable in the long term. Recruits are encouraged to learn that letting the team down for a short amount of time is better than compromising long-term service in the military.

Participant 3 (Group 2): *Awareness, talk to them, make them understand. Make them aware that even if they are in pain, even if they are injured, it is okay. Make them aware that the army will not stop operating if they are injured, and that as individuals, as soldiers, they are worth more than … Their presence for the next 5, 10, 15, 20, 25 years is more important than their presence at the next drill.*

Participant 2 (Group 1): *The long-term. And that’s the thing, is I try to explain to the candidates, because I have gone through injuries in my career, and I’ve had those setbacks. And trying to explain that OK, it’s OK to be injured right? Like yes, it sucks, and yes it makes you feel useless … Not contributing to the team. But like, the military is not going anywhere right now … there’s going to be another course.*

#### The military’s relationship with pain is a double-edged sword

The military culture around pain and injuries is challenging and presents a double-edged sword. On one side is the necessity of mission success and the ability to complete tasks in dangerous settings, while on the other is the potential for long-term harm to individuals.

The instructors mention that a considerable contributor to this double-edged sword is that military personnel are trained to push through pain to ensure operational readiness and protect their country. According to them, any deviation from this mind-set could potentially undermine mission success and overall military effectiveness.

Participant 2 (Group 1): *… unfortunately yes, the beliefs around pain are the way they are, but we can’t sacrifice – I don’t think that we can change that culture. Like in the military aspect. I think it would do more damage if we were to change our culture around pain.*

*… So, we’re viewed as a last resort, right? Essentially, but that last resort is useless if you have a bunch of soldiers that believe: “I’m in pain, so I don’t have to do my job.”*

*No, you’re in pain, and you’ve got to keep going. Because your country is depending on you. You have a* *mission to do, and that needs to get done. If we ever try to stray away from that belief “OK, it’s OK to be hurting. And you can get help right away.” No. It’s OK to be hurting, and you need to finish doing your job regardless. I* *think that if we stray from that, then our whole culture as a* *military, and our dynamic of mission success. Which is essentially every military’s goal around the world. If we stray away from that, it’s going to cause more damage than good.*

Instructors also note important downsides to this mind-set, as many soldiers push through pain even when facing severe injuries and often avoid seeking medical attention. The mind-set they develop prevents them from recognizing when medical care is necessary. While this mind-set is considered essential, instructors acknowledge that it comes at a cost.

Participant 2 (Group 1): *But I also think that like, it’s very much a double-edged sword. Where like … you get in that mind-set and then you struggle to turn it off right? So, like for myself, I’m injured right now, pretty severely. And it’s because of my mind-set of just go, go, go. Like it’s just pain. It’s just pain. Turn it off …*

Instructors also note that teaching by example has positive and negative aspects. As mentioned in subtheme 1.2, these examples help recruits understand their roles and how they should push themselves to overcome mental and physical barriers. However, these examples can also lead recruits to mimic behaviors such as pushing through injuries and avoiding the healthcare system.

Participant 2 (Group 1): *Yeah, and I felt like we’re a little bit blind to the fact that if we think that our actions – they look up to us, and that’s the truth. They all try to replicate the instructors in their own specific ways, right? … they try to do what we do right? So, in the event that oh, I’ve seen an instructor get injured. Like they all saw me go into pain, and grunt. And then just keep going right? And then they’re like “Well, if the [Rank] is doing it then I can do it too.” And I think that’s something that is what it is. Through just seeing, and maybe it’s a flaw in the system? But I also think that it’s a good thing. To be able to see that OK, this does happen, and you can push through. But it could also be negative in terms of like “OK, I’m injured and the [Rank] is pushing through.” But like … I think it can be (a double-edged sword) right? Like in some ways it can be. In other ways it can be good. I think that it’s very case by case basis.*

#### The organization of medical care within the military is a barrier to finding solutions

The instructors described a complex environment where the decision to seek medical help has many implications that are often not well communicated and can result in unexpected course failure for recruits.

Participant 2 (Group 1): *Like I had it a couple days ago. Where a candidate got two days of sick leave. So, they got put off course, and they missed mandatory periods. And they were like “Well, why am I off course?” “Well, you’re on leave. Like you’re missing periods?” And they’re like “Well they didn’t tell me that.” And I was like “But did you ask? Did you ask what the impacts are?” Right? You’ve got to be able to advocate for yourself …*

According to the instructors, one of the issues is that the medical system is independent from the training school. This organizational separation is designed to ensure that healthcare professionals can prioritize the health of recruits. However, this separation can have many negative consequences since the professionals in the medical system may not be aware of all the intricacies of the training courses and the impact that their decisions can have on the success of recruits.

Participant 2 (Group 1): *We don’t have our own medical system within the school. We use another unit … They don’t understand the training plan. They don’t understand the schedule or anything like that.*

*…*

Participant 3: *It’s purposeful because then, like every base is like that. The purpose behind that is so that we can’t push them to do things that they don’t want to do.*

The instructors mention that this leads the two systems to sometimes work against each other. For instance, instructors aim to develop resilience in recruits, while the medical system may inadvertently provide recruits with ways to avoid certain parts of the training, especially when instructors try teaching recruits to push through pain.

Participant 3 (Group 2): *Yes. But they have different mandates. Our mandate is to train soldiers, their mandate is to make sure the member is taken care of. If the member says he’s in pain, well, their mandate is to make sure the member is pain-free so he can continue training. Sometimes they have to give crutches since they can’t put any weight on their foot, well, that’s their mandate. Their mandate is a bit counter-productive …*

Participant 1 (Group 2): *The MIR works against us a little in terms of pain resilience. Because we try to make them understand: listen, it’s normal, this or that will happen, you’re going to have pain, be careful, deal with it. But as soon as they go to the MIR, and that’s what I understand about frustration, they’re told: “ah, you’re right to have pain, ah my God that’s not good, it’s not normal, ah rest, stop everything, stop everything, you’re hurting, stop everything.”*

For the instructors, there is also a perceived inconsistent application of medical restrictions, which can lead to confusion and resentment among recruits.

Participant 2 (Group 1): *So, we were in the field a couple courses ago where a candidate got a chit (doctor’s note) that allowed him to not carry a rucksack, but he was able to carry a day bag. Which allowed him to continue his training, and then somebody was like “Oh, my back hurts. I’m going to go to the medical center and get the same chit.” And then they went to the medical center, and they did not get the same chit. Because it’s case by case, and whatever circumstances led, their chit did not give them the permission to wear a rucksack or a day bag. So now you can’t continue your training, and you’re re-coursed.*

## Discussion

The current study aimed to gain a more comprehensive understanding of what pain beliefs are emphasized by basic training instructors during the initial phase of military training and how these relate to the broader cultural context of the CAF. We identified the intended messages conveyed during training, the culture surrounding pain in both basic training and the broader military, and critical reflections on how pain is addressed within the training environment and the military as a whole. Overall, our work underscores the tension between the military’s operational goals and the need for long-term health preservation. Our findings provide valuable insights into the challenges faced within the CAF regarding pain beliefs.

Pain and the culture surrounding it in the military have been studied from multiple perspectives, such as sociological, anthropological, and psychological. For instance, Harper^56^ puts forward the idea that experiencing pain is a necessity in the military and should be faced with stoicism and concealed from others. MacLeish^57^ discusses how the ability to ignore pain can become essential in deployment and come to be relied upon by military personnel. Cogan and collaborators^58^ highlight that military personnel avoid seeking medical aid as it is perceived as a sign of weakness in the military culture. Our results complement these perspectives by foregrounding the views of basic training instructors and by explicitly connecting these views to the concept of “pain beliefs” in the field of pain. Specifically, our findings help clarify how pain beliefs are discussed, understood, and transmitted by instructors, including their rationale for teaching them and their awareness of the impacts these beliefs can have on both recruits and themselves.

### Pain beliefs within the broader CAF

Parts of our results highlight some well-known phenomena surrounding pain in the military, but from the perspective of BMQ instructors. For instance, a recent study focusing on the broader experience of pain among CAF members/Veterans and their families reports that instructors taught CAF recruits to push through pain and used slogans such as the ones presented in our results.^16^ The same study also reports a hierarchy where the mission comes first and the self last.^16^ Lorber and Garcia^23^ similarly reported an account where a soldier learned to “suck it up and drive on” when facing pain. The results from these two studies^16,23^ suggest that the beliefs about pain described by the instructors in our study are similar to those of active CAF members and Veterans, indicating that these findings may reflect broader shared pain beliefs and experiences across the CAF.

In the current study, the instructors described a cultural pressure to persevere through pain that is compounded by the stigma surrounding medical care during their own service. A similar phenomenon was reported in a study with CAF recruits, which found that the culture and stigma surrounding injuries, as well as treatment-seeking behavior, contribute to poor health outcomes during basic training.^59^ The perspective of recruits complements the perspective of the instructors, suggesting that the instructors’ experience aligns with the experiences of recruits. Interestingly, a study found that resilience training may contribute to unintended pain-related consequences, since most military course failures in the CAF are due to overuse injuries (56%) rather than physical traumatic injuries (44%).^60^ The instructors in the current study did not seem aware of how prevalent overuse injuries were during courses or of the extent to which they may contribute to injuries and course failure among recruits through resilience training.^60^

The cultural pattern of persevering through pain and injuries could contribute to chronic pain in Veterans, as a recent qualitative study with CAF Veterans living with chronic pain found that a culture centered on mission importance and silent obedience encourages service members to endure pain.^61^ The participants in this qualitative study described how expectations to push through pain or injuries, along with the stigma attached to being injured, created fears of failure and of losing career opportunities.^61^

### Pain beliefs in other populations

Pain beliefs in other populations can help us better understand some of our results. Our findings showed that instructors reported that injuries often need to be visible to be acknowledged or validated by others in the military. In the general population, invalidation is associated with the underreporting of pain, which impedes assessment and treatment.^62^ These results by Boring and collaborators^62^ suggest that military personnel may receive poor assessment and treatment due to pain invalidation as part of the military culture.

Interestingly, some of the pain beliefs reported by the instructors in our study are also described by civilians. For example, pushing through pain is also seen in other populations, such as farmers who work through pain during high-demand seasons and nurses who continue working despite worsening pain.^63,64^ Similarly, the idea that injuries can be experienced as personal failures is also seen in civilian populations, such as dancers who perform despite injuries and setbacks, for whom being sidelined due to injury is associated with guilt, a sense of personal failure, and a threat to identity.^65^ The tendency observed in military personnel to continue functioning despite injury is similar to what is reported in a narrative review, which describes how the athletic identity is associated with a reluctance to report injuries and a willingness to play through them, illustrating how different forms of identity may influence pain beliefs in a similar way.^66^ Thus, the belief that pain must be endured, even when injuries are present, can arise outside the military. In particular, it could emerge in contexts where completing the task is viewed as more important than attending to pain or the body and where the person’s identity is strongly associated with completing the task, much like the way a mission is prioritized in the military. Perhaps the organizational structures surrounding these professions share similar cultures or beliefs regarding health, which could be investigated in future studies.

### The military identity

Our results highlighted the role that military identity plays in the development and maintenance of pain beliefs. According to Hart and Lancaster,^67^ the identity of military personnel becomes intertwined with the broader team identity, which can lead to identity fusion, in which a person’s identity becomes bound to being part of a team. These findings from Hart and Lancaster^67^ help contextualize why it may be so difficult for CAF members to care for themselves and address injuries, because once identity fusion has occurred, self-care may be perceived as coming at the expense of the team. Our results clarified the instructors’ perspective on this issue, particularly their understanding of the difficulties CAF members may face when seeking medical care, such as fears of letting others down or being removed from a team to which they feel strongly connected. This threat of being removed from the team appears to be grounded in actual military practices, as Noyek and collaborators^16^ reported that individuals perceived as “broken” were often excluded or sidelined by their team. Consistent with this, a study found that injuries that suddenly end military careers can lead to abrupt identity disruption.^68^ Moreover, the habits developed during military service may persist afterward and lead Veterans to continue pushing through pain to accomplish tasks.^61^ Taken together, the results of the studies presented in this paragraph suggest that the pain beliefs promoted by BMQ instructors, as identified in our study, may be internalized through identity fusion and may negatively influence the identities and post-service behaviors of service members.

An important point made by a participant in Majid and collaborators’^68^ study on CAF Veterans with chronic pain is that military personnel are broken down during basic training to form a military identity, but after service there is nothing in place to help them adapt this identity to the world outside the military. Considering this statement and our results, it would be pertinent to explore the potential for an intervention aimed at countering the negative aspects of the pain beliefs built into basic military training.

### The tensions surrounding pain beliefs

Despite the multiple challenges regarding pain beliefs presented in the current study, the CAF has begun addressing some of these challenges by fostering cultural changes aimed at creating a more supportive and accommodating environment.^69,70^ However, attempting to change how pain is discussed and treated reveals tensions within the military’s approach to pain. These tensions are discussed explicitly in subtheme 3.2 and are also alluded to implicitly across several themes and subthemes in the current study.

The first tension is found between recruits’ and instructors’ differing norms and expectations surrounding physical training. In our results, we saw that according to the instructors, recruits are not used to being sore or experiencing pain after a workout, perhaps because of a lack of experience and exposure. This lack of experience in physical training clashes with the military culture of building resilience and operational readiness. Together, these findings from our study suggest that some civilian views of pain and training are directly at odds with the instructors’ aim of instilling a culture of perseverance through pain. This tension may be alleviated as recruits’ beliefs surrounding pain shift and their self-identity starts to merge with a military identity.

The second tension is found between military and medical cultures, as the military prioritizes operational effectiveness and career advancement, whereas the medical system prioritizes pain reduction and rest when injured. In our results, we saw that the instructors discussed how the aims of the medical system could directly compete with their goals of resilience training, creating a constant cultural tension between the medical system and the military that may affect both recruits and instructors. For instance, we saw that this unresolved tension affected the instructors themselves, who often found it difficult to let others down or delay a promotion because of persistent pain conditions. However, the instructors recognized both the importance of their mandate in building resilience for military effectiveness and the unintended consequences that resilience training could have on health and wellness. Future research is needed to better understand how this unresolved cultural tension, and its associated emotional labor, is lived and experienced by BMQ instructors or other service members.

Overall, the findings of the current study contribute to a better understanding of a generally fragmented literature on pain beliefs in the military, a topic that has not yet been examined explicitly or in detail. Our results converge with observations in other populations, mainly active service members and Veterans.^16,21,61,68,71^ This convergence demonstrates continuity of pain norms across roles and highlights instructors as an important mechanism through which these beliefs are taught and sustained. Our results suggest that pain beliefs in the military are influenced by two broad currents that are sometimes at odds with each other: the official goals of the instructors and the unofficial culture surrounding pain in the military. On one hand, instructors have a clear goal and purpose concerning pain beliefs, aiming to prepare recruits for active duty and missions. This involves teaching recruits to push through pain to ensure mission success. On the other hand, the CAF has a deeply ingrained culture of dismissing injuries and their treatment. The instructors’ focus on preparing recruits for active duty and the CAF culture surrounding injury and pain management connect to broader issues beyond training, including operational readiness and the challenges Veterans can face when moving into civilian life.^6,72–75^ The multiple cultural tensions surrounding pain beliefs also help explain why changing pain beliefs and help-seeking attitudes within the military can be difficult.

### Practical implications

Overall, our results highlight that, according to basic training instructors, the pain beliefs present within the military are both necessary and potentially harmful for individuals. Our findings suggest several actionable points for military training and healthcare frameworks. First, military institutions might consider enhancing training on recognizing and differentiating between pain and injury within basic training curricula, as our findings suggest that the current training framework could benefit from further refinement. This training could be delivered by MIR staff, who are qualified to educate both recruits and instructors on these issues. Such education would help recruits better understand their physical limits, potentially reducing preventable injuries, while also equipping instructors to more effectively support recruits in identifying and addressing injuries. Additionally, this training could provide an opportunity to clarify how injuries may lead to being re-coursed, reducing the uncertainty surrounding visits to the MIR and encouraging recruits to seek medical assistance when necessary.

Enhancing the integration of healthcare within military training programmes may further reduce the tension between resilience training and seeking support. This improvement could help recruits feel more comfortable seeking medical assistance without fearing that it will compromise their success in the course or their position within the group. Strengthened collaboration between medical personnel and instructors, along with clearer protocols for managing injuries during training, might improve outcomes by balancing immediate resilience-building needs with long-term health priorities. Emphasizing that seeking medical attention is part of resilience could reduce stigma, encouraging recruits to maintain operational health more effectively while also preparing them for sustainable military careers.

Additionally, practical efforts should consider the adjustment process for service members transitioning to civilian life. Interventions addressing deeply ingrained beliefs surrounding pain endurance and injury avoidance should aim to facilitate the adoption of more adaptive attitudes toward medical care and recovery while simultaneously preserving Veterans’ identity and sense of pride in their military service. For healthcare professionals, specialized training could improve their understanding of the unique perspectives on pain and resilience that active service members and Veterans may hold. Tailored communication strategies and educational initiatives emphasizing the importance of seeking treatment without stigma might facilitate recovery and better support long-term health outcomes. Collaborating across military and healthcare systems to address these challenges could ensure continuity of care and promote overall well-being throughout service and beyond.

### Strengths, limitations and implications for future research

In our study, the perspectives from both Anglophone and Francophone instructors allowed for a greater breadth of experiences and viewpoints. While a potential limitation of focus groups is that some participants with views that do not fit the group may remain silent or feel uncomfortable sharing their opinions,^76^ the opposite appeared to happen in our study. Participants shared deeply personal experiences and were eager to disagree when their opinions diverged.

The current study primarily focused on instructors’ perspectives, which, while invaluable, may not fully capture how recruits internalize pain beliefs and apply them both during training and in later military service. The current study represents the perspective of some instructors and cannot be generalized to all instructors and military institutions. Future research should consider longitudinal and multicenter studies that follow recruits from initial training through active service, allowing for a more comprehensive view of how beliefs about pain and resilience evolve. Future research could investigate how instructors came to teach pain-related beliefs, including whether these beliefs were shaped by cultural norms, repeated teaching patterns, or formally included in the course content. Another potential avenue for building on the present findings is to further investigate how basic training instructors shape the pain beliefs described in our study, with attention to the specific teaching practices, corrections, modeling, and informal interactions through which pain beliefs are reinforced or challenged. Our results also briefly highlighted a current of change within CAF culture and training practices that could be explored in future studies. It should also be noted that messaging surrounding pain beliefs may differ under routine conditions versus periods of heightened threat, rapid mobilization, or deployment-focused operations. Future projects could investigate these contextual differences.

All participants were recruited from the main training site of the CAF in Saint-Jean-sur-Richelieu in Quebec, Canada, and may not represent the views of training instructors from the other training site in Borden, Ontario, Canada.^77^ The perspective of Canadian instructors may also not reflect the views of training instructors in other armed forces from different countries. Future studies could explore differences in training, culture, and beliefs across other training centers around the world.

Sex and gender differences play an important role in pain experiences and pain chronification.^78,79^ Our sample consisted of only men. Notably, prior studies report that masculinity permeates military culture and contributes to the perception of injuries as a weakness.^58,80^ A narrative review reports that this masculine tendency to perceive pain as weakness may persist among Veterans and lead to complications and refusal of treatment in older age.^81^ Because these three studies^58,80,81^ focused on men and masculinity, similar to our study, it would be valuable to explicitly explore whether this sentiment is echoed by women in the military. A study of women Veterans partially answers this question by reporting that women may experience discrimination and difficulty being accepted in a culture dominated by masculinity.^82^ However, the exact role of pain beliefs remains unclear, requiring dedicated research with women Veterans, instructors, recruits, and active service members.

While the study was grounded in the CAF context, military cultures vary widely across countries, especially regarding medical support systems, attitudes toward resilience, and risk management. Research in diverse military settings could clarify whether the findings represent universal aspects of military training or are specific to the Canadian context. Examining the long-term effects of the initial formation of pain beliefs on chronic injury rates and mental health post-service could provide insights into how military training environments impact Veterans’ health outcomes. Further research could help clarify the uncertainty regarding the impacts of seeking medical support in the military.

## Conclusion

The results of the current study provide valuable insights into the perspective of basic training instructors regarding the formation of pain beliefs within CAF basic training and detail how early military training embeds a culture prioritizing resilience, endurance, and mission success, often at the expense of individual health. While resilience training and the ability to push through pain are essential for operational readiness, the cultural expectations surrounding pain can lead to unintended consequences. By encouraging recruits to push their physical and mental limits, training may contribute to a culture of underreporting injuries, overlooking health needs, and stigmatizing medical care as a sign of weakness. When pain becomes linked to personal and team identity, recruits may avoid seeking medical attention, fearing they will be seen as failing their team or jeopardizing their position in the training programme. While these themes were observed in a military setting, similar experiences may occur in other populations. Finally, the current study highlights the struggle between meeting military objectives and maintaining long-term health, as recruits and military personnel often face difficult decisions regarding pain and injuries, which can deeply affect their career, health and identity.

## Supplementary Material

Supplemental Digital Content.docx
