# Supplementary material for: Pain Beliefs in the Military: A Qualitative Study Exploring the Perspectives of Instructors Within the Canadian Armed Forces on How Pain Is Addressed in Basic Training and the Broader Military Culture
Source: Can J Pain. 2026 Jul 28;10(1):2682925. doi: 10.1080/24740527.2026.2682925 (PMC13418701; doi:10.1080/24740527.2026.2682925)

**Supplemental Digital Content**

[**COREQ checklist with additional study details** 2](#_Toc189405346)

[**GRIPP2 Short Form with additional study details** 7](#_Toc189405347)

[**Topic guide for semi-structured focus groups** 8](#_Toc189405348)

[**Focus Group Handout** 11](#_Toc189405348)

[**Coding scheme** 15](#_Toc189405349)

# **COREQ checklist with additional study details**

Adapted from: Tong A, Sainsbury P, Craig J. Consolidated criteria for reporting qualitative research (COREQ): a 32-item checklist for interviews and focus groups. *International Journal for Quality in Health Care*. 2007;19(6):349-357.

| **No. Item** | **Questions/Description** | **Reported Section and/or Additional Details (if applicable)** |
| --- | --- | --- |
| **Domain 1: Research team and reﬂexivity** |  |  |
| Personal Characteristics |  |  |
| 1. Interviewer/facilitator | Which author/s conducted the interview or focus group? | See Methods. |
| 2. Credentials | What were the researcher’s credentials? E.g., PhD, MD | Mael Gagnon-Mailhot: BSc, MSc.  Peter Stilwell: BKin, DC, MSc, PhD.  M. Gabrielle Pagé: BA, MA, PhD.  Derek Speirs: CD, Living with pain; pain advocate.  Hélène Le Scelleur: M. Soc. Serv, Living with pain; pain advocate.  Timothy H. Wideman: PT, PhD. |
| 3. Occupation | What was their occupation at the time of the study? | Mael Gagnon-Mailhot: PhD Student.  Peter Stilwell: Postdoctoral Researcher.  M. Gabrielle Pagé: Assistant Professor.  Derek Speirs: Retired  Hélène Le Scelleur: Vice-chair for the Advisory Council for Veterans at the Chronic Pain Centre of Excellence and PhD Student.  Timothy H. Wideman: Associate Professor. |
| 4. Gender | Was the team member a man, woman, non-binary or other? | Mael Gagnon-Mailhot: Man.  Peter Stilwell: Man.  M. Gabrielle Pagé: Woman.  Derek Speirs: Man.  Hélène Le Scelleur: Woman.  Timothy H. Wideman: Man. |
| 5. Training and experience | What relevant training or experience did the researcher have? | Mael Gagnon-Mailhot: Qualitative methods training/experience.  Peter Stilwell: Qualitative methods training/experience, including past work in phenomenology.  M. Gabrielle Pagé: Mixed methods training/experience, clinical psychologist.  Derek Speirs: Veteran, personal pain experience, pain advocate.  Hélène Le Scelleur: Veteran, personal pain experience, pain advocate.  Timothy H. Wideman: Mixed methods training/experience, physiotherapist. |
| Relationship with participants |  |  |
| 6. Relationship established | Was a relationship established prior to study commencement? | No relationship was established prior to study commencement. |
| 7. Participant knowledge of the interviewer | What did the participants know about the researcher? e.g. personal goals, reasons for doing the research | Participants were given the study aim of better understanding pain beliefs in the military. Participants were also informed that our hope was that this study may ultimately inform the design of tools and approaches to help healthcare practitioners better recognize and address Veterans’ pain and its negative life-impacts. Further, during the introduction of the group discussions, additional details were provided regarding the team, such as professional backgrounds (e.g. T.H.W. is a physiotherapist). |
| 8. Interviewer characteristics | What characteristics were reported about the interviewer/facilitator? e.g. Bias, assumptions, reasons and interests in the research topic | See item 7 above. |
| **Domain 2: study design** |  |  |
| Theoretical framework |  |  |
| 9. Methodological orientation and Theory | What methodological orientation was stated to underpin the study? e.g. grounded theory, ethnography, phenomenology | See Methods. |
| Participant selection |  |  |
| 10. Sampling | How were participants selected? e.g. purposive, convenience, consecutive, snowball | See Methods. |
| 11. Method of approach | How were participants approached? e.g. face-to-face, telephone, mail, email | See Methods. |
| 12. Sample size | How many participants were in the study? | See Results. |
| 13. Non-participation | How many people refused to participate or dropped out? Reasons? | See Results. |
| Setting |  |  |
| 14. Setting of data collection | Where was the data collected? e.g. home, clinic, workplace | See Methods. |
| 15. Presence of non-participants | Was anyone else present besides the participants and researchers? | See Methods. |
| 16. Description of sample | What are the important characteristics of the sample? e.g. demographic data, date | See Results. |
| Data collection |  |  |
| 17. Interview guide | Were questions, prompts, guides provided by the authors? Was it pilot tested? | See Methods, Results and semi-structured guide found in this document. |
| 18. Repeat interviews | Were repeat interviews carried out? If yes, how many? | No. |
| 19. Audio/visual recording | Did the research use audio or visual recording to collect the data? | See Methods. |
| 20. Field notes | Were ﬁeld notes made during and/or after the interview or focus group? | See Methods. |
| 21. Duration | What was the duration of the interviews or focus group? | See Results. |
| 22. Data saturation | Was data saturation discussed? | See Methods/Results. Please note that we used the nuanced concept of “information power” due to known issues with the concept of saturation. See Malterud et al., (2016) in the manuscript references. The image following this table contains additional details regarding our information power analysis. |
| 23. Transcripts returned | Were transcripts returned to participants for comment and/or correction? | No, but transcripts were reviewed by researchers present during the interviews. |
| **Domain 3: analysis and ﬁndings** |  |  |
| Data analysis |  |  |
| 24. Number of data coders | How many data coders coded the data? | See Methods. |
| 25. Description of the coding tree | Did authors provide a description of the coding tree? | See Methods and coding scheme in this document. |
| 26. Derivation of themes | Were themes identiﬁed in advance or derived from the data? | See Methods and Results. |
| 27. Software | What software, if applicable, was used to manage the data? | See Methods. |
| 28. Participant checking | Did participants provide feedback on the ﬁndings? | See Methods regarding real-time member checking. |
| Reporting |  |  |
| 29. Quotations presented | Were participant quotations presented to illustrate the themes/ﬁndings? Was each quotation identiﬁed? e.g. participant number | See Results. |
| 30. Data and ﬁndings consistent | Was there consistency between the data presented and the ﬁndings? | See Results. |
| 31. Clarity of major themes | Were major themes clearly presented in the ﬁndings? | See Results. |
| 32. Clarity of minor themes | Is there a description of diverse cases or discussion of minor themes? | See Results and Discussion. |


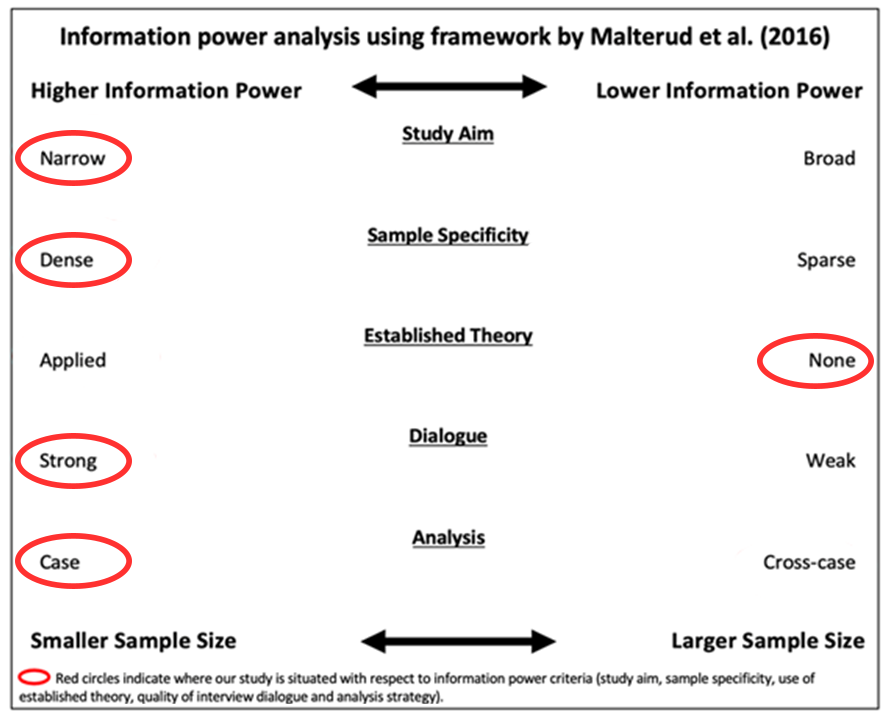


# **GRIPP2 Short Form with additional study details**

Adapted from: Staniszewska S, Brett J, Simera I, Seers K, Mockford C, Goodlad S, et al. GRIPP2 reporting checklists: tools to improve reporting of patient and public involvement in research. *BMJ*. 2017;358:j3453.

| **Item** | **Reported section** |
| --- | --- |
| Report the aim of PPI (patient and public involvement) in the study. | PPI was used to ensure the study was shaped by Veterans’ lived experience of chronic pain and by the realities of military culture. The aims of involving partners were to: (1) refine the study focus and interview questions so they reflected meaningful issues for Veterans; (2) help ensure our data collection methods (demographic questions, focus group guide) contained accurate and current military terminology; (3) strengthen the trustworthiness of our analysis with comparison to their lived experience; and (4) improve relevance and clarity of the manuscript for clinical, military, and Veteran audiences. |
| Provide a clear description of the methods used for PPI in the study. | Veterans living with chronic pain were recruited through the Quebec Pain Research Network (QPRN). Veteran collaboration occurred at multiple stages with different levels of involvement:  Project conception and overall study design (n = 4 Veterans): D.S., H.L.S. and two additional Veteran collaborators contributed to shaping the study focus, framing the topic, and advising on feasibility considerations for engaging basic training instructors.  Recruitment approach and study procedures (n = 4 Veterans): D.S., H.L.S. and the same two additional Veteran collaborators provided feedback on recruitment procedures and practical considerations for conducting the work in a military training environment.  Focus group discussion guide development (n = 3 Veterans): D.S., H.L.S. and one additional Veteran collaborators worked with the research team to develop and refine the semi-structured discussion guide, ensuring the questions captured relevant cultural norms, training expectations, and the ways pain and injury are talked about and managed in military contexts.  Interpretation of findings (n = 2 Veterans): D.S. and H.L.S. contributed to interpretation by reviewing the themes and illustrative quotations and discussing how the findings aligned with lived experience in basic training and military service. Their input supported the credibility of interpretations and helped support that the themes were communicated in ways that were meaningful to Veterans and sensitive to military context.  Manuscript development (n = 2 Veterans): D.S. and H.L.S. reviewed the developing narrative of findings and provided input on emphasis and clarity.  They contributed primarily through structured team meetings and follow up feedback. They contributed primarily through structured team meetings and follow up feedback. H.S.L. was compensated for their time and contributions. |
| Outcomes—Report the results of PPI in the study, including both positive and negative outcomes. | See Methods, Results and Discussion. Partner (D.S.) was involved in all aspects of the study and (D.S and H.L.S.) shaped the findings. Positive outcomes included the partners highlighting areas to emphasize in the manuscript (e.g. the importance of unlimited liability and mission success for the military). No negative outcomes were apparent. |
| Outcomes—Comment on the extent to which PPI influenced the study overall. Describe positive and negative effects. | Partners (D.S and H.L.S.) played a large and essential role in the study. Positive effects included many in-depth discussions that helped shape and revise findings to be more impactful. No negative effects were apparent. |
| Comment critically on the study, reflecting on the things that went well and those that did not, so others can learn from this experience. | See Methods. Regular debriefs with partners (D.S and H.L.S.) were scheduled, and this went particularly well. We encouraged them to share how the preliminary findings aligned with their personal experiences and knowledge of others’ experiences through their extensive peer-support and advocacy work. This facilitated in-depth discussions that shaped the direction of the work. We established relationships with our partners before the study, which facilitated open and honest conversations. This requires time and financial investment (partners were offered compensation), which we feel is essential. |

**Topic guide for semi-structured focus groups with basic training instructors at the *Canadian Forces Leadership and Recruit School***

**Introduction:**

- Round table introductions of facilitators and participants.
  - Facilitators to clarify that they do not have a military background, but have been working to build their understanding of life in the military
- Circulate handout (see page 4 below).
  - Review Page 1 together
    - Discuss the specific aims of this discussion and connect this to the overarching aim of this line of research
      - Clarify that all forms of pain are of interest – including pain with injury, chronic pain, and pain without injury that is associated with a physically demanding task (long run, carrying heavy pack, sustaining difficult postures)
    - Clarify that there are no right or wrong answers, differences in opinions are welcomed and that more sharing will help us achieve our objectives
    - Remind participants of confidentiality, and that they can discontinue their participation at any point during the discussion (participants already received recruitment document and consented)
    - Ask for any questions
  - Review Page 2 together
    - Read excerpts from Joining Instructions

**Understanding the military state of mind and behaviour in relation to pain:**

- Can you please tell us more about the military state of mind and behaviour that you are trying to cultivate among new recruits? We are particularly interested in understanding this in relation to developing physical and mental endurance and resilience.
  - **Additional prompts:**
    - In what ways are you hoping that a civilian would change (in relation to resilience and mental/physical endurance) over the course of basic training?
- We’re interested in learning more about the specifics of the military state of mind and behaviour. Can you please tell me about how the Basic Military Qualification may help a new recruit to manage their pain in the context of a combat mission?
  - **Additional prompts**:
    - Imagine a new recruit has recently completed their Basic Military Qualification and is now in their first combat mission. Imagine that they have been instructed to quickly run from position A to B, but as they are completing this, they experience an intense muscle cramp. In this context, how would you hope that their basic training would help them manage this difficult scenario?
      - In this context, how might their basic training help them think differently about their pain?
      - In this context, how might their basic training help them respond to their pain differently?
      - In this context, how might their basic training help them act differently?
    - Can you tell us more about what you are hoping to inculcate in new recruits in relation to pain and/or its management during active service?

**Process of instilling and reinforcing a military culture in relation to pain:**

- Building on what we have already discussed, can you share *how* you, as instructors, help instill new recruits with a *military state of mind and behaviour* in relation to their pain?
  - **Additional prompts:**
    - Can you share specific strategies that you use or teach during basic training?
    - How do you or your colleagues shape new recruits’ understandings of pain?
    - How do you or your colleagues shape new recruits’ behaviors when they are in pain?
- How do you think this military culture around pain is reinforced after basic training? Can you provide specific examples?

**Phrases, sayings and mottos:**

- Can you share any (additional) pain-related phrases, sayings or mottos that you’ve come across and/or that may have been used in the past, in your work as a trainer?
  - **Additional prompts**:
    - Circulate image of marines and discuss motto: *pain is weakness leaving the body*.
    - Share other sayings that we have come across: *If you can feel the pain, you’re still alive* ; *Shut the fuck up and take the pain!*
- If any phrases, sayings or mottos are endorsed:
  - How do you think these ideas about pain could help you and others serve in the military?
    - **Additional prompts:**
      - Unpack the meanings and intended function of the messages discussed.
      - Is messaging intended to facilitate specific understandings of pain?
      - Is messaging intended to facilitate certain behavior/coping?
- Try to exhaust the positive/functional value of these ideas within the military.

**Exploring potential downsides of military on pain (during service):**

- Injuries are often part of military service. What happens when someone has a painful injury, and it is not safe to continue to serve? What is the military culture on pain in this context?
- (Recap everything that was shared about military culture on pain and the value that this has for service – do not recap what instructors do to instill this).
- Clearly there are a lot of benefits of this culture around pain to facilitate active service. Does anyone see any potential gaps or downsides of the military culture on pain?
  - **Additional Prompt:**
    - Are there any areas for improvement in basic training that could help fill these gaps?
- **Wrap up:**
  - Is there anything else anyone would like to share related to military culture and pain?
    - Additional Prompt:
      - Any other general thoughts on this topic, or any thoughts specific to basic training or active service?
- Thank participants and close the session.

**Focus Group Handout for Participating Basic Training Instructors**

**Study Title:** Understanding pain beliefs in the military and their role in shaping identity and coping among Veterans living with pain.

**Facilitators:**

- Timothy Wideman, PhD, McGill University
- Gabrielle Pagé, PhD, Université de Montréal

**Specific objective of this discussion**: Understand how new recruits are trained to manage their pain during active service.

**Clarification regarding pain:** All forms of pain are of interest to this discussion. This includes (1) pain that results from a new injury, (2) pain that persists longer than the expected healing time of an injury, and (3) pain without any injury that is associated with a physically demanding task, such as a long run, carrying a heavy pack, and/or sustaining a challenging posture.

**Overarching objective of this line of research:** To improve care for Canadian Veterans that are suffering with persistent pain and struggling with their transition to civilian life.

**Reminders:**

- There are no right or wrong answers.
- Differences in opinions are welcomed.
- The more you share, the more likely these findings can be used to help Veterans living with pain.
- This discussion is confidential and you can stop participating at any time.


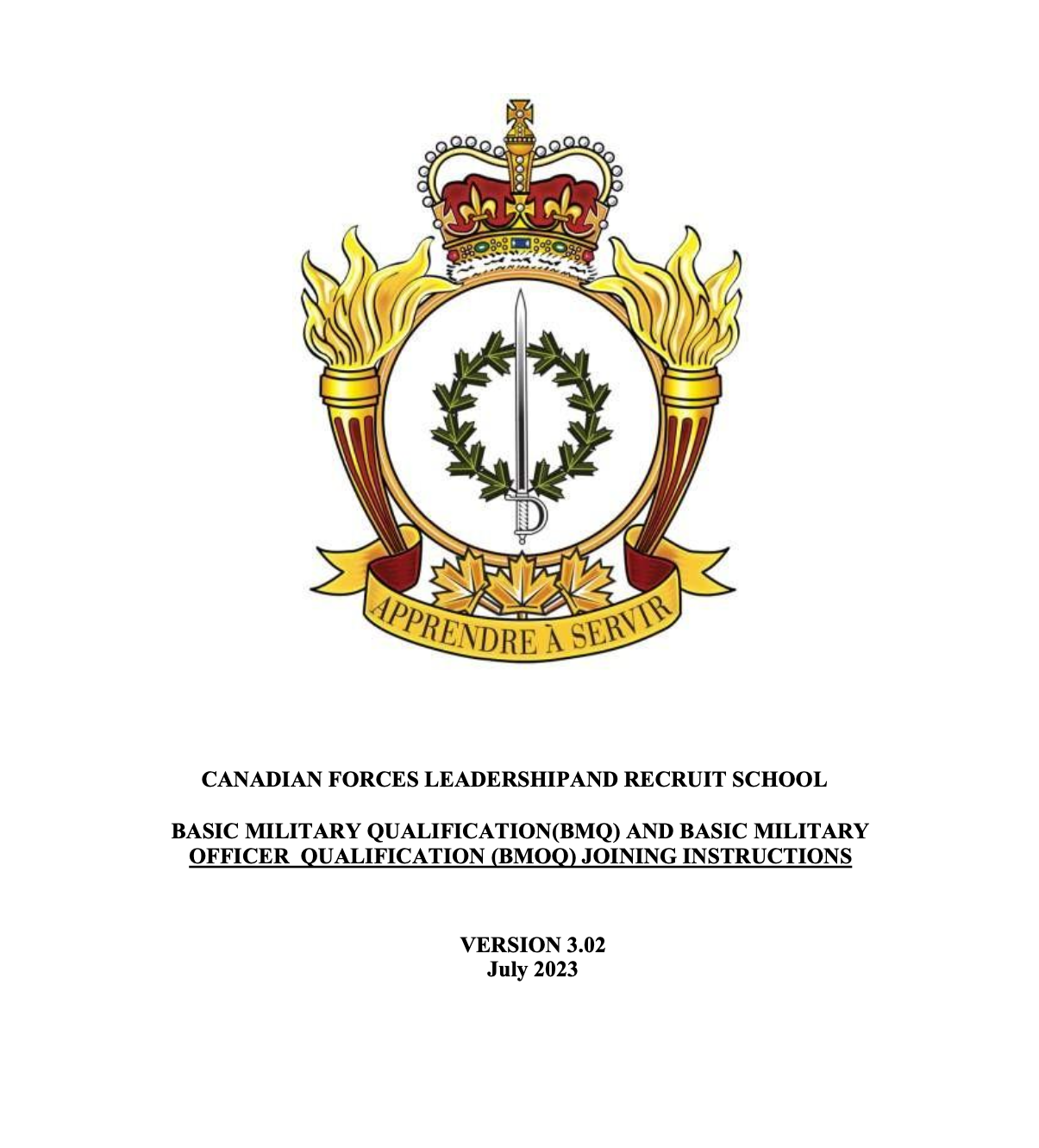


**Excerpts from page 5 of the Joining Instructions, Version 3.02**

**CFLRS Mission** (Bolded emphasis added)

1. The Canadian Forces Leadership and Recruit School’s (CFLRS) mission is to shape, instruct and inspire candidates using innovative training methods geared towards resilience and transformational leadership **to prepare them for their professional service in the Canadian Armed Forces (CAF).**

**Basic Training Description** (Bolded emphasis added)

5. Basic training provides the knowledge that is common to all trades and elements. It develops a **military state of mind,** professional conduct, **mental and physical endurance**, and the skills necessary for the profession of arms. The training is both physically and mentally demanding; prepare yourself according

**
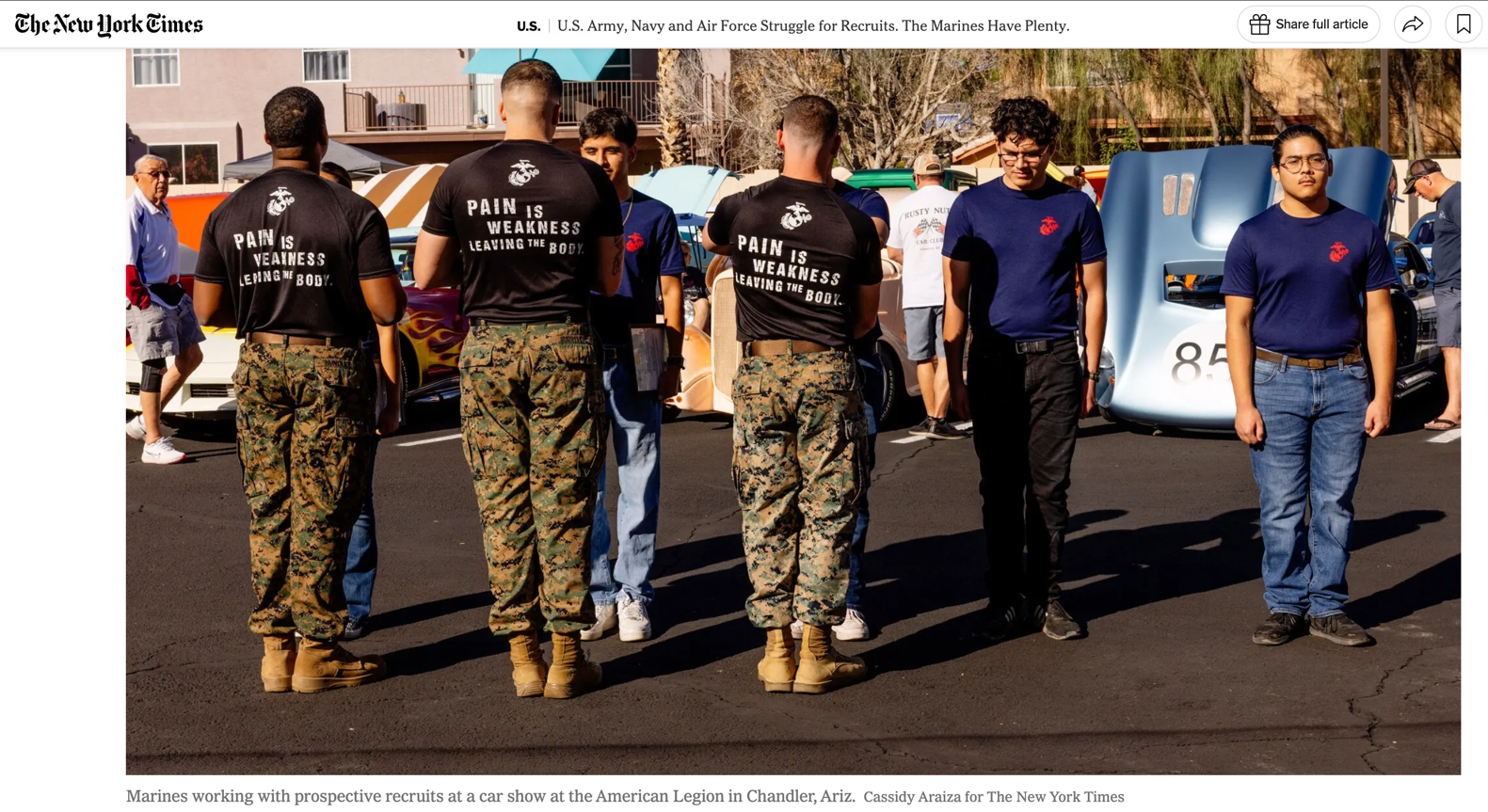
**

# **Coding scheme**


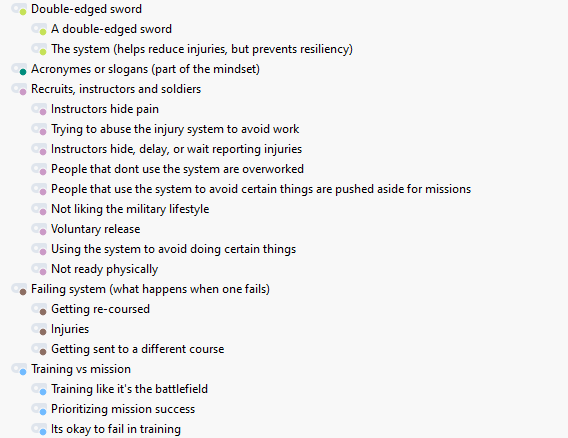

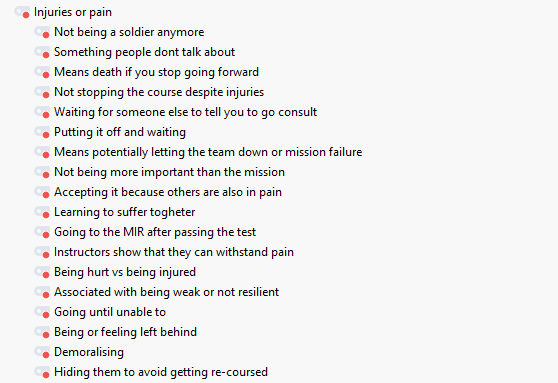

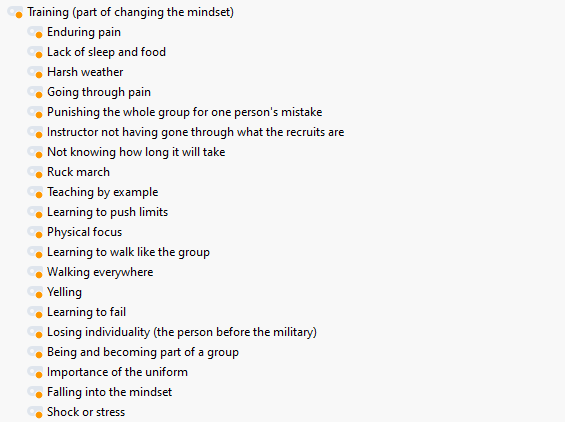

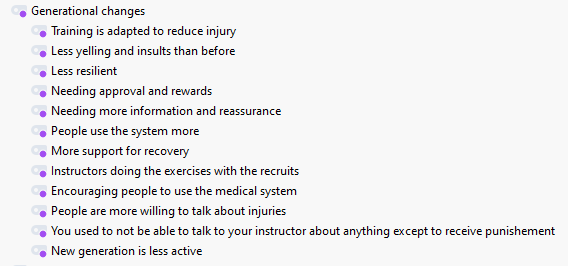

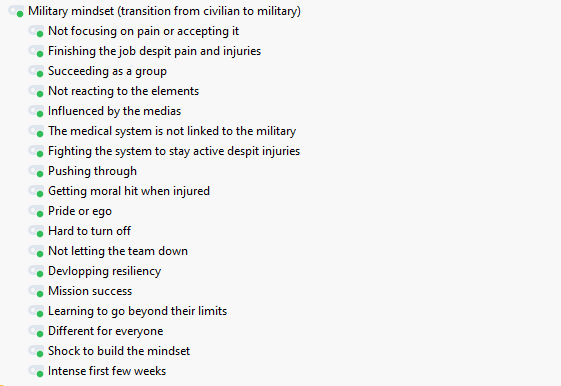

Supplement: Supplemental Digital Content.docx [file UCJP_A_2682925_SM4356.docx]
